# Patients’ experiences of the COVID-19 pandemic and the change to telephone consultations in cancer care

**DOI:** 10.1007/s00520-022-07390-y

**Published:** 2022-10-15

**Authors:** Hanne Bødtcher, Katrine Vammen Lindblad, Dina Melanie Sørensen, Elizabeth Rosted, Eva Kjeldsted, Helle Gert Christensen, Mads Nordahl Svendsen, Linda Aagaard Thomsen, Susanne Oksbjerg Dalton

**Affiliations:** 1 Science to Society, Danish Cancer Society Research Center, Copenhagen, Denmark; 2 Survivorship and Inequality in Cancer, Danish Cancer Society Research Center, Copenhagen, Denmark; 3 Department of Clinical Oncology and Palliative Care, Zealand University Hospital, Naestved, Denmark; 4 Department of Clinical Oncology and Palliative Care, Zealand University Hospital, Roskilde, Denmark; 5 Department of Regional Health Research, University of Southern Denmark, Odense, Denmark

**Keywords:** COVID-19, Cancer care, Telephone consultation, Patients’ experiences, Qualitative study

## Abstract

**Purpose:**

During the COVID-19 pandemic, teleconsultations have increasingly been used to reduce physical contact and thus risk of infection. This study investigated how patients with cancer experienced the COVID-19 pandemic and how they perceived the change from in-person consultations to telephone consultations in an oncology outpatient clinic. The aim was to provide insights that could optimize the future use of teleconsultations in cancer care.

**Methods:**

This qualitative study included 15 patients with colorectal, breast, gynecological, lung, or prostate cancer treated at the outpatient clinic at the Department of Clinical Oncology and Palliative Care, Zealand University Hospital, Denmark in June or July 2020. Data were collected through semi-structured individual interviews and analyzed by thematic analysis.

**Results:**

Patients with cancer experienced social, psychological, and organizational consequences of the COVID-19 pandemic related to their cancer care. Not all patients were comfortable with telephone consultations. Six themes were identified: (1) double burden as a consequence of simultaneous cancer and the COVID-19 pandemic, (2) parameters for patient satisfaction with telephone consultations, (3) the importance of relatives attending consultations, (4) loss of information and nuances during telephone consultations, (5) the impact of physicians’ language and communicative skills during telephone consultations, and (6) patients’ suggestions for future telephone consultations.

**Conclusion:**

Beyond the COVID-19 pandemic, it is important that hospitals offering teleconsultations involve patients’ preferences, consider for which patients and consultations the solution is suitable, which technology to use, how to prepare patients and relatives, and how to provide physicians with the necessary communicative skills.

## Background

Due to the SARS-CoV-2 (herein called COVID-19) pandemic, healthcare systems worldwide had to change their daily practices to limit the outbreak in the society and, at the same time, continue safe and effective patient care [1]. Patients with cancer are particularly susceptible to infections [2] and seem to be at higher risk of COVID-19-related admissions to the intensive care unit and mortality [3–5]. Social distancing has been one of the restrictions used to limit the pandemic, and health care organizations have replaced in-person consultations with for instance telephone consultations [6].

Telemedicine is the use of telecommunication technology as a tool to deliver health care where physical access to care is limited [7]. It has been employed in cancer care for years, but the use accelerated significantly during the COVID-19 pandemic [6]. A systematic review shows that telehealth interventions in cancer care represent a convenient approach, which can potentially reduce treatment burden and disruption to cancer survivors’ lives [8]. Tele-oncology interventions have demonstrated both clinical value and cost-effectiveness, as well as high levels of patient satisfaction [7].

Only a few studies have investigated how patients with cancer experience the COVID-19 pandemic. A Canadian qualitative study found that the COVID-19 outbreak was a major medical, psychological, social, and occupational stressor for women with breast cancer [9], and similar results were found in studies with other cancer diseases [10–13]. A Danish mixed method study found that during the second wave of the COVID-19 pandemic, patients with cancer had in general, low levels of distress and high levels of resilience, and family support was invaluable in critical times [14].

There is limited knowledge regarding how patients with cancer experience telephone consultations during the COVID-19 pandemic. Results from qualitative studies indicate that some patients with cancer experienced telephone consultation as rushed and found it difficult to discuss their treatment issues and that telephone consultations required patients to be more persistent in order to obtain necessary information [15, 16]. Digital consultations were by some patients perceived as impersonal, relationships established prior to the teleconsultation, supported patients to engage with healthcare professionals during teleconsultations, and patients often preferred in-person consultations [17–19].

This study explored how patients with cancer experienced the COVID-19 pandemic and the change to telephone consultations during the first wave of the COVID-19 pandemic in an oncology outpatient clinic in Denmark. The aim was to provide insights that could optimize the future use of teleconsultations in cancer care.

## Methods

### Setting and design

The study took place at the Department of Clinical Oncology and Palliative Care, Zealand University Hospital, Denmark. The department provides all oncological treatment in Region Zealand, of about 0.8 million inhabitants. In Denmark, cancer care is tax-financed and provided free of charge to all patients. Due to the first wave of the COVID-19 pandemic, the department changed outpatient appointments with a physician to telephone consultations from 15 March 2020. (Video consultations were only offered on experimental basis.) Exceptions to this change were consultations with newly diagnosed patients where the treatment plan had to be decided or changed or situations where the physician considered in-person consultation necessary. The physician decided 14 days prior to the consultation whether an in-person visit was warranted, informed the medical secretaries to schedule either in-person or telephone consultation, and inform the patient about the change to telephone consultation and time for the call. The secretary scheduled 30 min per telephone consultation. In order to reduce the number of visitors in the clinics, relatives were not allowed to participate in in-person consultations or treatment sessions. A survey showed that two-thirds (68%) of patients had at least one telephone consultation from 15 March 2020 to 30 April 2020 [20]. The study was a qualitative study with semi-structured individual interviews.

### Participants and procedures

We used purposeful sampling to ensure variation in gender, age, and cancer diagnosis and selected 15 patients from the hospital’s electronic outpatient lists. Inclusion criteria specified that the patients had at least one physician appointment converted to a telephone consultation, were 18 years or older, and were Danish speaking. Exclusion criteria were the inability to give consent. Patients were invited to and informed about the study by telephone in June and July 2020, and they received an information letter afterwards. Interview appointments were made with patients willing to participate. Due to COVID-19 restrictions, the interviews were conducted as telephone interviews, and participants gave oral informed consent at the beginning of the interview. Three of the authors (HB, KLV, DMS) mediated five patient interviews each. The interview lasted 15–45 min. The study was approved by the Region Zealand Data Protection Agency (number REG-076-2020) and did not require Ethics Committee approval.

### The interview guide

In April 2020, a clinical quality assurance project was conducted where two of the authors (DMS, EK) asked ten randomly selected patients with breast, lung, or urological cancers about their perspectives on the introduction of telephone consultations. Their experiences were used to develop the semi-structured interview guide for this study. The interview guide included basic questions on patient and cancer-specific characteristics such as cancer type, patient trajectory, educational level, employment status, cohabitation status, relatives, distance to the hospital, and means of transportation. Other questions concerned patients’ experiences with having cancer, cancer treatment, contact to the oncology department, telephone consultations, and involvement in cancer treatment of relatives during the COVID-19 pandemic.

### Data analysis

Interviews were audio recorded and transcribed in verbatim by two of the authors and an undergraduate student. Transcripts were checked against the original audio recordings for accuracy. One interview was excluded from the analysis due to the poor quality of the audio recording. Data were analyzed using thematic analysis as described by Braun and Clarke [21] with the following six phases: phase 1 “Familiarizing yourself with your data,” phase 2 “Generating initial codes,” phase 3 “Searching for themes,” phase 4 “Reviewing themes,” phase 5 “Defining and naming themes,” and phase 6 “Producing the report.” Three of the authors completed phases 1–3 (HB, KLV, DMS). They listened to the recorded interviews, read the transcripts, generated the initial coding individually in the software program NVivo 1.3, compared and discussed the coding until consensus was reached, and sorted the different codes into potential themes. Phases 4–6 were completed by four of the authors (HB, KLV, DMS, ER). Themes were reviewed and refined in relation to the entire data set, and afterwards, defined and named.

## Results

### Characteristics of the participants

Participants’ median age was 60 years (range 52–86), with equal gender distribution. The majority lived with a partner (86%), had mandatory school (7–9 years of primary and lower secondary school) or vocational education (64%), had retired (64%), had less than 60 min transportation time from their home to the hospital (79%), and reached the hospital by car either by themselves or with a relative (93%). The participants had breast, colorectal, gynecological, lung, or prostate cancer. The majority (64%) were in the follow-up phase of their cancer trajectory, and 36% were in active treatment.

### Themes identified

Six themes were identified: (1) double burden as a consequence of simultaneous cancer and the COVID-19 pandemic, (2) parameters for patient satisfaction with telephone consultations, (3) the importance of relatives attending consultations, (4) loss of information and nuances during telephone consultations, (5) the impact of physicians’ language and communicative skills during telephone consultations, and (6) patients’ suggestions for future telephone consultations.

### Double burden as a consequence of simultaneous cancer and the COVID-19 pandemic

Patients experienced psychological, social, and organizational consequences of the COVID-19 pandemic. They perceived their cancer diagnosis and the COVID-19 pandemic as a double burden and double insecurity.

“Overall, it is a double insecure situation to be in when you are a cancer patient in Corona times. That is the way it is in general.”

“The fact that I have two diagnoses, and that I have the Corona situation on top of it, that has made it a confusing and stressful period.”

Patients expressed general worries related to the cancer disease, choice of treatment, risk of side effects, and fear of recurrence in combination with concerns related to COVID-19. They reported that the pandemic limited family visits, and due to their vulnerability towards COVID-19, some patients isolated themselves at home, which affected their mood. Patients in treatment or follow-up experienced insecurity, anxiety, and complications induced by cancellation or postponement of consultations, treatment, or rehabilitation. Women with breast cancer expressed concerns about potential delayed detection of cancer recurrence as they were used to physical examinations of the breast at in-person consultations. Other patients did not experience that the COVID-19 pandemic influenced their cancer treatment at all.

### Parameters for patient satisfaction with telephone consultations

The patients understood the need for changing from in-person consultations to telephone consultations due to the COVID-19 pandemic, and they acknowledged the differences between the two consultation forms. At the same time, they reported that the healthcare professionals encouraged them to contact the department if they had unanswered questions. The patients also expressed that a telephone consultation was better than no consultation.

“They encouraged me to contact them because they are aware that something can be missed this way. So I actually think you have had a good feeling that issues are being taken care of.”

Overall, the participants expressed satisfaction with the telephone consultations if a number of parameters were in place. Patients expressed satisfaction if the physician called at the agreed time, had set aside sufficient time for the consultation, provided adequate information, asked relevant questions, problems were solved, it was easy to get in contact with the department, and it was possible to have physical examinations if needed.

“I am completely satisfied because I got the basic information I needed, and they told me what they could see on the scan, so no problems there. But as I said, I would have preferred to talk face-to-face. However, no problem with the information I got, I got the information I probably needed and that is all right.”

### The importance of relatives attending consultations

Patients perceived the emotional, cognitive, and practical support they received from their relatives as essential. During ordinary in-person consultations, relatives took part in preparing questions for consultations, participated in consultations, and assisted the patient with remembering information provided during consultations. However, patients who used to have a relative accompanying them for in-person consultations often did not have a relative with them for telephone consultations, and lacking the presence of a relative was perceived as disturbing. Reasons for relatives not attending the telephone consultations were mostly practical such as the physician not calling at the scheduled time, relatives not being physically present with the patient, the call being handled during working hours, or the patient not being aware of the opportunity. Some patients had relatives on speaker during the telephone consultation, which worked well.

“It has been awful. It really has! Because he could not support me. There has been many times where I have been unwell in the beginning. And there I really needed my husband.”

Patients considered telephone consultations less important than in-person consultations. A reason was that the patients assumed that the content of a telephone consultation was less critical if the hospital did not encourage participation of a relative.

“Well, when you have an appointment at the hospital because there is a scan result, they ensure that you are coming, and you have the opportunity to bring a relative, if it is a critical test result you get. So when I could not bring my wife to those meetings because of Corona, then I assumed that the test results were not so critical.”

### Loss of information and nuances during telephone consultations

Some patients experienced receiving insufficient information during telephone consultations, which induced insecurity. Some patients refrained from asking questions during telephone consultations, whereas during in-person consultations, they were inspired to ask additional questions. Patients perceived telephone consultations as more factual, shorter, and with fewer nuances. Moreover, they missed the possibility of seeing scan images for a better understanding of the disease.

“I got the information that the physician believed was most important. That is for sure. It is certain that the person, who called me, told me, what was important to know. I have no problem with that. The problem is that I might have thought of something else if I sat and talked with the person, a real person.”

“At my conversation or consultation I just had the impression that the goal was to tick me off. She just asked if I was fine and if I had the medicine I needed and then she was ready to hang up. Therefore, I got the impression that was just it. It did not motivate me to ask questions or say anything.”

Telephone consultations were perceived as more impersonal due to lack of eye contact and body language. Patients needed facial expressions of the physician as indications of whether the physician was busy, and to anticipate, whether the physicians’ message was good or bad. Furthermore, the patients felt that the physician was unable to read their reaction to the provided information and that they could hide reactions.

“When we evaluate images and so on, we can see at the physician whether there are tears of joy or is it now real.”

“I want to see the physician’s expression, how she says it and tells me about things. And they can immediately see my reaction.”

### The impact of physicians’ language and communicative skills during telephone consultations

How patients perceive the telephone consultations depended partly on the physician’s communicative skills. It was more challenging for patients to understand physicians with a foreign accent at telephone consultations, due to the lack of nonverbal communication. In addition, patients felt that it was more difficult for physicians with a foreign accent to verify if they understood the given information. Some patients found it difficult to hear what was said on the telephone.

“I have been at the oncology department at an ordinary in-person consultation and it was also a [nationality is mentioned] but then we had the body language, and then it is much easier. The physician see if I do not understand it – then he/she just tries again. That is not possible over the telephone.”

“Maybe it depends on which physician you talk to. How good are they at communicating, at managing a telephone consultation. That is what it depends on and not on the content of the conversation…. By being open minded, asking some questions, when you have a dialogue where you discuss various issues, that you may not be aware of, but through their competencies and expertise ask about.”

### Patients’ suggestions for future telephone consultations

The patients mentioned the advantages of telephone consultations such as the reduced risk of COVID-19, saved transportation time, easier planning related to work, and less stress. They also expressed that telephone consultations were most suitable at follow-up, when the cancer disease was uncomplicated, for short or practical information, positive test results, and when the patient and physician already knew each other. In-person consultations were preferred to discuss treatment plan or introduction of new interventions. Some patients did not consider telephone consultations suitable for patients with cancer at all. Few patients had experienced a video consultation with, for instance, their general practitioner, and they described advantages such as the possibility to see facial expressions, scan images, or showing, for example, skin rashes. Video consultations were considered technically more challenging than telephone consultations and with the same lack of physical examinations and detachment introduced when communicating through a technical device.

For future use of telephone consultations, the patients suggested to optimize the consultations by supporting patients’ expectations and preparations. Patients should be encouraged to write down questions and relevant information during the consultation and to have relatives attending on speaker. The patients suggested a standard agenda for telephone consultations to ensure that relevant subjects are discussed. Patients prefer talking to the same physician throughout their cancer trajectory, and they requested a guarantee that the first consultation was in-person.

“So I think that it could be improved by having an in-person consultation before you start the treatment course. Or also that you got a call where they say: “We will call you that day, and from the hospital’s point of view it is important that we talk about this and that, and you are welcome to think about what is currently on your mind.” Because otherwise it is often the case that in a stressful situation with cancer and treatment and such issues you forget half of the questions you have.”

## Discussion

The aim of this qualitative interview study was to explore how patients with cancer experienced the COVID-19 pandemic and how they perceived the change from in-person consultations to telephone consultations to provide insights for the improvement of teleconsultations in the future. Patients experienced a double burden as a consequence of cancer and the COVID-19 pandemic occurring simultaneously; they reported parameters important for a successful telephone consultation and stated the importance of relatives attending consultations. The patients reported that telephone consultations implied a loss of information and nuances and emphasized the significance of physicians’ language and communicative skills. In order to improve future teleconsultations, patients requested information to help patients prepare for the consultation. Furthermore, they suggested having relatives attending on speaker, that the first consultation is an in-person consultation, the patients have same physician throughout the cancer trajectory, and that telephone consultations are primarily used for follow-up, when the cancer disease is uncomplicated, or for short and practical information.

Patients in this study experienced negative psychological consequences of cancer and the COVID-19 pandemic. This parallels findings from a Danish cross-sectional study, which showed that 80% of patients with cancer were concerned about getting COVID-19, and this concern was associated with lower quality of life and emotional functioning [22]. Relatedly, a survey conducted in 16 European countries found that 71% of patients with gynecological cancer were concerned about cancer progression if their treatment or follow-up was canceled or postponed during the pandemic [23]. Cancer as the primary health threat and worries about cancer treatment delays were also revealed in other qualitative studies [13, 19]. Dieperink et al. found that patients undergoing active chemotherapy, recent surgery, were older and had comorbidities felt especially vulnerable [14]. Taken together, these results indicate that health care professionals need to be aware of the psychological distress that patients with cancer may experience during a crisis such as the pandemic or when a planned treatment is postponed or canceled.

While patients acknowledged the advantages of telephone consultations such as the reduced risk of infection and saved transportation time, most patients in our study preferred in-person consultations. This parallels findings from a Danish survey, which found that only 36% of patients with urological cancer were positive towards replacing in-person consultations with telephone consultations [24], while 67% of patients with hematological cancer were positive towards telephone consultations [25]. The acceptance of telephone consultations increased with higher levels of education. Kjeldsted et al. found that having breast cancer, anxiety, low health literacy, or being in follow-up after treatment were associated with less positive experiences with teleconsultations [20]. The specific cancer disease and the physical or emotional challenges caused by the disease such as altered body image and psychological distress in women with breast cancer [26] may influence the way they experience telephone consultations. In our study, some patients expressed contradictory attitudes towards the use of telephone consultations. Their perceptions and experiences may have been different if they had been better prepared for the change and had been involved in the decision. Healthcare professionals need to be aware of how patient and disease characteristics may influence the preference and how patients perceive teleconsultations.

In our study, patients considered telephone consultations less important than in-person consultations as relatives were not invited. Healthcare professionals need to be aware of this when planning teleconsultations. Additionally, it is important that healthcare professionals recognize that relatives’ participation in consultations is important to patients and, therefore, should be encouraged for telephone consultations. Patients in the present study considered telephone consultations impersonal, which is consistent with other research [8, 17]. During telephone consultations, patients were less likely to ask questions and were highly dependent on the physician’s communicative skills, as the ability to use eye contact, body language, physical examination, and images was unavailable. It is therefore important that hospitals choosing to use teleconsultations consider for which patients the solution is suitable, which technology to use, how to prepare patients and relatives, and how to provide physicians with the necessary communicative skills.

### Strengths and limitations

Patients in this study represented various cancer types, had a wide age span, and gender was equally represented. The fact that few patients had medium or higher level of education and only few patients lived far from the hospital or needed public transportation could have affected the attitude and preferences for telephone consultations. Most patients in our study had limited experiences with telephone consultations, and the experiences discussed in the present paper represent the first wave of the COVID-19 pandemic, where patients and healthcare professionals were not accustomed to the use of telephone consultations. Patients were interviewed by telephone to reduce COVID-19 risks. This may have influenced the length, the responses, the depth, and nuances in the interviews compared to in-person interviews. Future qualitative studies may include a larger sample to provide more breadth and in-depth knowledge and perspectives from patients with different cancer sites, treatment-related side effects as well as at different points in the treatment trajectory on the transitions to digital consultations by telephone or video in cancer care.

## Conclusion

Healthcare professionals need to be aware of the psychological distress patients with cancer may experience due to the COVID-19 pandemic or when planned treatment is postponed or canceled. Patient and disease characteristics as well as patient preferences affect how patients perceive telephone consultations. Beyond the COVID-19 pandemic, it is important that hospitals offering teleconsultations involve patients’ preferences, consider for which patients and consultations the solution is suitable, which technology to use, how to prepare patients and relatives, and how to provide physicians with the necessary communicative skills.

## Data Availability

The data that support the findings of this study are available on request from the corresponding author. The data are not publically available due to privacy or ethical restrictions.
